# Normalization of a conversation tool to promote shared decision making about anticoagulation in patients with atrial fibrillation within a practical randomized trial of its effectiveness: a cross-sectional study

**DOI:** 10.1186/s13063-020-04305-2

**Published:** 2020-05-12

**Authors:** Gabriela Spencer-Bonilla, Anjali Thota, Paige Organick, Oscar J. Ponce, Marleen Kunneman, Rachel Giblon, Megan E. Branda, Angela L. Sivly, Emma Behnken, Carl R. May, Victor M. Montori, Shared Decision Making for Atrial Fibrillation (SDM4AFib) Trial Investigators, Victor Montori, Megan E. Branda, Juan Pablo Brito, Marleen Kunneman, Ian Hargraves, Angela L. Sivly, Kirsten Fleming, Bruce Burnett, Mark Linzer, Haeshik Gorr, Elizabeth Jackson, Erik Hess, Takeki Suzuki, James Hamilton, Peter A. Noseworthy, Haeshik Gorr, Alexander Haffke, Mark Linzer, Jule Muegge, Sara Poplau, Benjamin Simpson, Miamoua Vang, Mike Wambua, Joel Anderson, Emma Behnken, Fernanda Bellolio, Renee Cabalka, Michael Ferrara, Rachel Giblon, Jonathan Inselman, Annie LeBlanc, Peter Noseworthy, Marc Olive, Paige Organick, Nilay Shah, Gabriela Spencer-Bonilla, Amy Stier, Anjali Thota, Henry Ting, Derek Vanmeter, Claudia Zeballos-Palacios, Park Nicollet-HealthPartners, Carol Abullarde, Lisa Harvey, Shelly Keune, Timothy Smith, Shannon Stephens, Bryan Barksdale, Theresa Hickey, Roma Peters, Memrie Price, Connie Watson, Douglas Wolfe, Gordon Guyatt, Brian Haynes, George Tomlinson, Paul Daniels, Bernard Gersh, Thomas Jaeger, Robert McBane

**Affiliations:** 1 Knowledge and Evaluation Research Unit, Mayo Clinic, 200 1st Street SW, Rochester, MN 55905 USA; 2 Department of Medicine, Stanford University School of Medicine, Stanford, CA USA; 3 CONEVID (Unidad de Conocimiento y Evidencia), Universidad Peruana Cayetano Heredia, Lima, Peru; 4 Medical Decision Making, Department of Biomedical Data Sciences, Leiden University Medical Center, Leiden, Netherlands; 5 Division of Health Care Policy and Research, Department of Health Sciences Research, Mayo Clinic College of Medicine, Rochester, MN USA; 6 Colorado School of Public Health, University of Colorado Denver Anschutz Medical Campus, Denver, CO USA; 7 Faculty of Public Health and Policy, London School of Hygiene and Tropical Medicine, London, UK

**Keywords:** Atrial fibrillation, Shared decision making, Trials, Normalization process theory, Burnout, Anticoagulation, Trial procedures, Conversation aid

## Abstract

**Background:**

Shared decision making (SDM) implementation remains challenging. The factors that promote or hinder implementation of SDM tools for use during the consultation, including contextual factors such as clinician burnout and organizational support, remain unclear. We explored these factors in the context of a practical multicenter randomized trial evaluating the effectiveness of an SDM conversation tool for patients with atrial fibrillation considering anticoagulation therapy.

**Methods:**

In this cross-sectional study, we recruited clinicians who were regularly involved in conversations with patients regarding anticoagulation for atrial fibrillation. Clinicians reported their characteristics and burnout symptoms using the two-item Maslach Burnout Inventory. Clinicians were trained in using the SDM tool, and they recorded their perceptions of the tool’s normalization potential using the Normalization MeAsure Development (NoMAD) survey instrument and verbally reflected on their answers to these survey questions. When possible, the training sessions and clinicians’ verbal responses to the conversation tool were recorded.

**Results:**

Our study comprised 183 clinicians recruited into the trial (168 with survey responses and 112 with recordings). Overall, clinicians gave high scores to the normalization potential of the intervention; they endorsed all domains of normalization to the same extent, regardless of site, clinician characteristics, or burnout ratings. In interviews, clinicians paid significant attention to making sense of the tool. Tool buy-in seemed to depend heavily on their ability to see the tool as accurate and “evidence-based” and their perceptions of having time in the consultation to use it.

**Conclusions:**

While time in the consultation remains a barrier, we did not find a significant association between burnout symptoms and normalization of an SDM conversation tool. Possible areas for improving the normalization of SDM conversation tools in clinical practice include enabling collaboration among clinicians to implement the tool and reporting how clinicians elsewhere use the tool. Direct measures of normalization (i.e., observing how often clinicians access the tool in practice outside of the clinical trial) may further elucidate the role that contextual factors, such as clinician burnout, play in the implementation of SDM.

**Trial registration:**

ClinicalTrials.gov, NCT02905032. Registered on 9 September 2016.

## Background

Implementing complex interventions, such as use of shared decision making (SDM) tools, within clinical encounters requires participation from patients and clinicians. Despite evidence of their effectiveness and enthusiastic policymaker endorsement, the real-world uptake of tools to promote SDM has been limited [1]. Process evaluations of practical or effectiveness trials can contribute to understanding how complex interventions, such as use of SDM tools, may be implemented beyond the trial period [2].

Normalization Process Theory (NPT) provides a framework for understanding the process of implementing complex interventions in healthcare [3, 4]. NPT proposes that complex interventions (e.g., SDM tools in practice) become routinely embedded (implemented and integrated) in their organizational and professional contexts as a result of people working, individually and collectively, to implement them. It involves four domains: (1) making sense of the intervention, (2) getting people involved, (3) doing the work, and (4) evaluating the intervention in daily practice (Table 1). NPT can be used as a framework to understand how healthcare interventions interact with the existing clinic processes, clinical practice, and patient–clinician encounters and the work professionals do to enact them [3, 4].

**Table 1:** Domains of Normalization Process Theory

NPT domain	Focus
Coherence	Making sense of the proposed intervention
Cognitive participation	Getting people involved in the implementation project, buy-in
Collective action	Doing the work to make the intervention part of daily practice, organizational resources, training, and division of labor; confidence, expertise, and intervention workability
Reflexive monitoring	Evaluating the use of the intervention in daily practice and monitoring its value

*NPT* Normalization Process Theory

Although the barriers and facilitators for the implementation of patient-facing decision aids have been reported, less is known about the factors that promote or hinder the implementation of SDM conversation aids, which are used by the patient–clinician dyad during the clinical encounter [5]. The implementation of SDM tools during the consultation involves the execution and orchestration of individual and collective work and the work that is promoted or hindered as integration into routine practice takes place. The persistent difference between enthusiasm for SDM and limited documented use of SDM tools in practice strongly suggests the presence of hindering factors (e.g., clinician burnout, lack of organizational support, logistical barriers). Here, we explore the factors that promote or hinder this work within an ongoing practical randomized trial requiring the integration of an SDM conversation tool in practice to evaluate its effectiveness against usual care. This SDM conversation tool, the Anticoagulation Choice Decision Aid, was designed to assist patients with atrial fibrillation and their clinicians with the decision of whether and how to use anticoagulants to prevent thromboembolic strokes [6]. We report on the participating clinicians’ characteristics and professional roles, levels of burnout, and perspectives on normalizing the use of the Anticoagulation Choice Decision Aid tool within the workflow of their clinical practices.

## Methods

### Setting and participants

The institutional review boards of the Mayo Clinic (approval 16-005409) and two other participating sites (Park Nicolette-HealthPartners and Hennepin Healthcare System) approved all study procedures. We recruited clinicians for a randomized trial (Shared Decision Making for Atrial Fibrillation [SDM4Afib]) evaluating the effectiveness of the Anticoagulation Choice Decision Aid tool versus usual care in patients with nonvalvular atrial fibrillation at risk for thromboembolic strokes and considering anticoagulation (ClinicalTrials.gov registration number NCT02905032) [7].

The tool, which can be found at anticoagulationdecisionaid.mayoclinic.org, was designed to support collaborative conversations between patients and clinicians to determine whether to use anticoagulation, given the patient’s risk of stroke, and how to anticoagulate (using either warfarin or direct oral anticoagulants) based on the relative merits (bleeding risk, dosing, need for monitoring, reversibility, cost, and interactions with foods and medications) of these options.

**Fig. 1:**
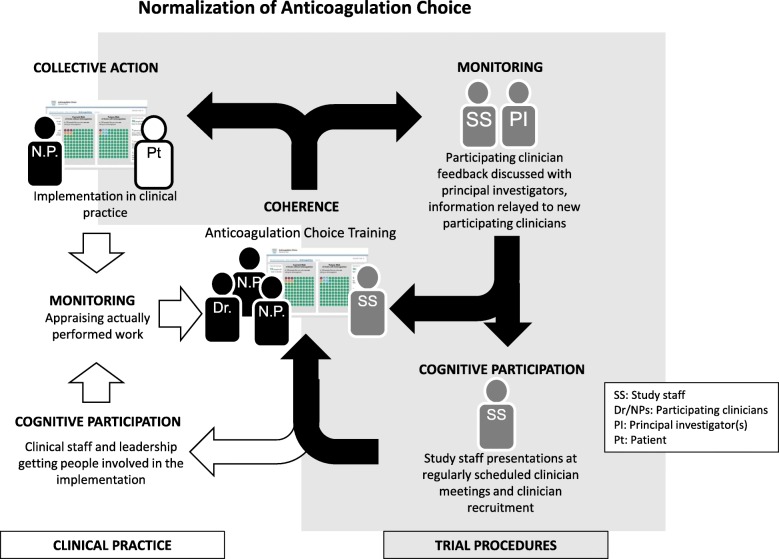
Normalization of Anticoagulation Choice Decision Aid tool

The SDM4Afib trial protocol [7] and the design of the Anticoagulation Choice Decision Aid tool have been described previously [6]. Briefly, we recruited clinicians from three hospital sites in Minnesota: an academic medical center, a suburban community group practice, and a safety net health system. Clinicians were eligible if they were involved in conversations about anticoagulation with patients with atrial fibrillation. Eligible clinicians were invited to participate in the trial via e-mail and during established clinic meetings at primary care, emergency, cardiology, and dedicated anticoagulation practices at participating sites. After consenting to participate in the trial, the clinicians took part in a brief training session, either individually or in groups. In each session, study staff demonstrated how to use the Anticoagulation Choice Decision Aid tool with a patient through role playing with the clinician and, when feasible, by reviewing a brief video-recorded demonstration. Some of these training sessions took place “just in time” prior to appointments with eligible patients (which sometimes prohibited a more extensive training session with video recording), while others were scheduled outside the time of clinical duties. The clinicians were then asked to complete a questionnaire. During and after training sessions, participating clinicians were invited to explore issues of normalization of the SDM tool into their practice with prompts such as, “How does this tool differ from your everyday work?” and “What resources do you have for integrating this tool into practice?” Spontaneous and prompted comments made by clinicians during training sessions were recorded with their consent. Feedback and questions from training sessions and interviews were discussed with the study principal investigators. Recommendations from those conversations were shared with study staff via a live electronic database and monthly in-person/phone study staff meetings (Fig. 1, gray panel).

### Statistical analysis

All quantitative analyses were performed using SAS software (SAS Institute Inc., Cary, NC, USA). We used a chi-square test to assess the association between levels of burnout and site (academic medical center, community group practice, and safety net health system). We then used analysis of covariance (ANCOVA) to investigate differences between mean NoMAD domain scores and levels of burnout and callousness, adjusting for gender, site, years in practice, practitioner type, and specialty type (cardiology vs. other). *P* values < 0.05 were considered statistically significant.

### Assessing normalization: quantitative approach

The clinician questionnaire was used to collect information about demographic and professional characteristics, burnout symptoms, and clinicians’ perceptions of the Anticoagulation Choice Decision Aid’s normalization potential (Additional file 1).

Burnout was assessed using two items of the Maslach Burnout Inventory (MBI), “I feel burned out from my work” and “I have become more callous toward people since I took this job,” that have strong associations with the full MBI and key outcomes such as suicidality and desire to drop out of training [8]. The MBI items were scored on a 7-point scale where “never” contributed 0 points and “every day” contributed 6 points. Similar to previously reported methods, burnout was classified as “high,” “moderate,” or “low” [8–10]. High burnout was classified as feeling burned out from work or feeling more callous toward others once or more per week (4 points or greater). Medium burnout was having feelings of burnout or callousness once or more per month but less often than once per week (2 or 3 points). Low burnout was classified as feeling burned out and more callous toward others a few times per year or less (1 point or less).

Clinicians answered questions regarding their familiarity with the tool and the Normalization Measure Development (NoMAD) instrument after training [11]. Familiarity was rated on 10-point Likert scale anchored by responses such as “still feels very new” and “feels completely familiar” at the extremes. The NoMAD instrument elicits stakeholder views about how interventions impact their work and whether the new intervention could become normalized in their setting by probing the four domains of NPT (Table 1) [12, 13]. In our study, we omitted questions about “reflexive monitoring” because participants had not yet had a chance to integrate the SDM tool into their practice. The 15 NoMAD items, comprising 3 domains (5 items in each), were scored on a 5-point Likert scale, where “strongly agree” was assigned a score of 5 and “strongly disagree” was assigned a score of 1. Psychometric testing of this instrument supports taking the average score for all items within each domain [11].

### Assessing normalization: qualitative approach

Training across all sites captured feedback from 112 clinicians, 40 (36%) of whom received individual training and were interviewed one-to-one. We used a computer-generated simple random sample of 30 training recordings for a theory-led framework analysis. Proceedings were transcribed and classified using inductive coding into NPT domains (Table 1). The four coders (AT, GSB, OJP, and PO) calibrated their judgments by analyzing two recordings independently and successively, guided by a preliminary codebook. Disagreements were discussed, and the codebook was iterated accordingly with the addition of helpful examples. The remaining 28 recordings were analyzed independently in pairs, with disagreements discussed and resolved between reviewer pairs or by a third coder when necessary.

## Results

Table 2 reports the characteristics of 168 of the 183 enrolled clinicians by site with available survey data at the time of analysis. Except for questions related to burnout, response rates exceeded 90%. The academic medical center had fewer female clinicians and more cardiology specialists than the other sites, while the community group practice clinicians were mostly family physicians and included more clinicians in training (residents) than the other sites. Nonresident clinicians averaged 10 to 18 years in practice. About 20% of clinicians reported feeling burned out at least once per week, and 7% reported having become more callous at least once per week since taking their job. We found no significant associations between these traits and practice site (*P* = 0.28 and 0.34, respectively). Of these 183 clinicians who were trained and recruited, 112 were recorded.

**Table 2:** Clinician characteristics

Clinician characteristics	Academic medical center (*n* = 94)	Community group practice (*n* = 43)	Safety net system (*n* = 46)	Total (*N* = 183)
Missing information (*n*)	12	0	3	15
Women (*n*, %)	32 (39)	27 (63)	24 (56)	83 (49)
Age (mean, SD)	43 (12)	41 (12)	43 (11)	43 (12)
Clinician type (*n*, %)				
MD/DO	61 (74)	39 (91)	25 (58)	125 (74)
Resident	24 (29)	19 (44)	2 (5)	45 (27)
NP/PA	17 (21)	4 (9)	10 (23)	31 (19)
PharmD	4 (5)	0 (0)	4 (9)	8 (5)
RN	0 (0)	0 (0)	4 (9)	4 (2)
Clinician specialty (*n*, %)				
Family medicine	2 (2)	28 (65)	0 (0)	30 (18)
Internal medicine	10 (12)	6 (14)	32 (74)	48 (29)
Cardiology	52 (64)	5 (12)	7 (16)	64 (38)
Pharmacy	4 (5)	0 (0)	0 (0)	4 (2)
Other	14 (17)	4 (9)	4 (9)	22 (13)
Years in practice (mean, SD)^*^	13 (10)	18 (9)	10 (10)	13 (10)

^a^Excludes resident physicians (physicians in training)

### Normalization

In describing the possibility to integrate the SDM tool into their practices, 168 clinicians responded to the whole 0–10 scale with a mean of 6.6 (SD 2.5); cardiologists gave, on average, ratings that were 2 points higher. The mean NPT domain scores were 4.1 of 5 (SD 0.5) for coherence, 4.3 (0.5) for cognitive participation, and 3.9 (0.5) for collective action (Table 3). Using ANCOVA adjusted for clinician characteristics, there was no significant association between level of burnout and NPT domain scores or sites (results not shown).

**Table 3:** Normalization perceptions

Normalization perceptions	Academic medical center (*n* = 94)	Community group practice (*n* = 43)	Safety net system (*n* = 46)	Total (*N* = 183)
Missing (*n*)	12	0	3	15
SDM Integration: 1 (worst) to 10 (best); mean (SD)				
Feels familiar	7.2 (2.6)	5.9 (3.0)	5.8 (2.7)	6.5 (2.8)
Currently part of normal practice	6.1 (3.6)	5.3 (3.5)	5.2 (3.2)	5.7 (3.5)
Will become part of practice	7.7 (2.3)	7.5 (2.4)	7.4 (1.9)	7.5 (2.2)
NPT domains: agree/strongly agree (*n*, %)				
Coherence				
Differs from normal work	62 (76)	30 (70)	30 (70)	122 (73)
Shared understanding by staff	68 (83)	35 (81)	33 (77)	136 (81)
Understand how SDM affects my work	67 (82)	37 (86)	40 (93)	144 (86)
I see the value	76 (93)	39 (91)	39 (91)	154 (92)
Cognitive participation				
There are drivers	57 (70)	35 (81)	34 (79)	126 (75)
I believe it’s a legitimate part of work	73 (89)	39 (91)	37 (86)	149 (89)
Open to working with colleagues to improve	80 (98)	42 (98)	40 (93)	162 (96)
Will continue to support	78 (95)	39 (91)	38 (88)	155 (92)
Collective action				
Easy to integrate	69 (84)	31 (72)	30 (70)	130 (77)
Disruption of working relationships^a^	9 (11)	2 (5)	2 (5)	13 (8)
Confidence in others’ abilities	58 (71)	30 (70)	29 (67)	117 (70)
Assigned to people with appropriate skills	59 (72)	35 (81)	26 (61)	120 (71)
Sufficient training	57 (70)	29 (67)	33 (77)	119 (71)
Sufficient resources	64 (78)	30 (70)	30 (70)	124 (74)
Support from management	61 (74)	33 (77)	27 (63)	121 (72)

*NPT NPT* Normalization Process Theory, *SDM* Shared decision making

^a^Inverse coding used for items with negative framing

#### Coherence

When analyzing the recording proceedings, more than half (60%) of the 338 clinician comments or utterances fell within the *coherence* domain (i.e., understanding the purpose and role of the tool in care). Use of the tool was identified as differing from normal ways of working by 73% of surveyed clinicians. While they identified its visual component as an addition to their usual conversation, clinicians varied in terms of perceiving that it would fundamentally support a different conversation. Some clinicians stated that it was part of their obligation to discuss all of the issues related to anticoagulation and that they were already having this kind of conversation “on a regular basis.” As such, some were “afraid that it might not be much different” from what they already do. For example, a participating clinician explained:

*It’s basically a similar discussion to what I usually have. I think the graphical representation of what I’m discussing would be nice and probably [make it] easier.*

However, 154 (92%) clinicians agreed that they “saw the value” of using the Anticoagulation Choice Decision Aid tool and agreed that using it was a legitimate part of their work. They alluded to the visual aid as helpful in their efforts to help patients understand their risk of stroke. They also thought using the tool standardized the conversation about anticoagulation with at-risk patients:

*The way I think about this study is that it’s not comparing ‘shared decision making’ to ‘no shared decision making.’ It’s comparing standard care to a structured way of doing decision making. Because … we have conversations with our patients every single time, it’s just, it’s not structured, everyone does it differently. So, we’re comparing the usual way that we do it to using a structured tool.*

Of note, this was one of only two clinician statements among a total of 338 utterances that mentioned the term “shared decision making.”

#### Cognitive participation

Many comments coded under “cognitive participation” also pertained to “coherence” or “sense-making” work. Common issues that came up when clinicians negotiated “buy-in” for the tool pertained to the “risk of bleeding” and “reversibility.” While most clinicians agreed with statements that were consistent with high cognitive participation, sometimes they suggested content modifications that they would want made in order to optimize the tool for their personal use. For example, some clinicians questioned the accuracy of the bleeding estimates presented, requesting more granularity in the data presented in the tool:

*I would feel OK using it for now and explaining, to the patient, that yes, there is a bleeding risk but the risk of intracranial hemorrhage and of dying from a bleed is very small and is usually considered less [than what is presented in the tool].*

In some cases, clinicians suggested that their continued use of the tool was contingent on certain modifications to its content. For example, some requested that the tool include information about a new reversal agent for direct anticoagulants that was recently released. Others wanted the tool to reflect differences they believed were important between available direct oral anticoagulants, such as nuances in bleeding risk:

*I also think that it would be important to mention that there is one direct inhibitor that has been shown to have less bleeding risk, you know? … Which is apixaban, so patients need to know this too if you’re going to be full-disclosure, evidence-based!*

Almost all clinicians were open to working with colleagues in new ways to use the Anticoagulation Choice Decision Aid tool. However, 25% of clinicians surveyed did not agree that there were “drivers” or key people in leadership moving forward the implementation of the SDM tool in practice. While this was seldom explicitly mentioned by clinicians during training sessions, several of them inquired whether specific people at their institutions were participating in the trial and, unfortunately, were largely unaware of which clinicians and departments were using the tool.

#### Collective action

Although most clinicians endorsed coherence and cognitive participation, fewer endorsed statements representing collective action (i.e., having confidence in others’ abilities to use Anticoagulation Choice Decision Aid tool). Statements prompted by this question were often vague and difficult to interpret. Consultation time, however, was consistently and clearly described as a scarce yet necessary resource for using the tool in practice. Particularly, the following statement shows the interplay between collective action and cognitive participation. In this statement, the clinician alludes to time barriers and highlights how the effort required to participate in a trial and to implement the intervention being tested are conflated. When time was limited, especially in the context of a research study, the tool ceased to be a legitimate part of their daily work and instead was relegated to the position of a research “add on”:

*[If] the patient doesn’t show up until right on time or whatever, we’re just gonna have to say, “Well, this isn’t gonna work today.” So, ya know, you always have that option, so I think we want to be good citizens in the research community here, but, on the other hand, have to balance it with our … the personal needs of the individual patients.*

Comments on the “cost” also often encompassed both collective action and cognitive participation as clinicians negotiated whether it was their role to give patients information about the out-of-pocket cost of the available anticoagulation strategies as the tool was designed to do. Some identified the pharmacists as resources, while others cited lack of pharmacists with time to provide personalized cost information as a challenge:

*I’m curious ’cause I don’t have access to that in the ambulatory setting but the inpatient pharmacists do.… Before they even discharge people, they give that lovely thing that says, ‘Here’s their choices and how much it costs.’ … Would you be able to look into that? Or ask if that would be a resource?*

Clinicians also identified a lack of environmental resources for using the Anticoagulation Choice Decision Aid tool. Besides time, they cited that the rooms they used were not formatted so that both the clinician and patient could share a single computer screen. They also commented on the challenge of finding and accessing the tool online before each use, because the computers were shared between different healthcare professionals, and the tool was not integrated into the electronic medical record.

## Discussion

### Summary of findings

Embedding an intervention in clinical routines is necessary to evaluate its effectiveness in practice. This process takes individual and collective work subject to barriers and facilitators. Identifying and addressing these factors is critical in the design and implementation of practical clinical trials. This is particularly important when barriers and facilitators may differ across sites of a multisite trial. Here, we explored the embedding of an SDM intervention in routine clinical consultations as we recruited clinicians for a multicenter trial evaluating it. Clinicians rated the normalization potential of the intervention highly; for cardiologists, using the SDM tool in care made more sense than for other types of clinicians. Clinicians endorsed all domains of normalization to the same extent, regardless of site, clinician characteristics, or burnout or callousness ratings. Ninety-two percent of clinicians saw the value of using the Anticoagulation Choice Decision Aid tool, and 96% were open to working with colleagues to improve the use of it in practice. Fewer clinicians saw how the Anticoagulation Choice Decision Aid tool differed from their normal work (72%), agreed that there were drivers leading the implementation of the conversation aid (75%), or responded in agreement with items related to collective action (70–77%). Qualitatively, clinicians paid most attention to making sense of the tool. Tool buy-in seemed to depend heavily on clinicians’ ability to see the tool as accurate and “evidence-based” and on their having time to use it during consultations. They mentioned that the Anticoagulation Choice Decision Aid tool might lead to standardization of clinical encounters. Collective action issues focused on who has time and information to use the tool (e.g., enrolling pharmacists and the information to which they have access when including patient out-of-pocket costs in SDM conversations). Few clinicians commented on the concept of “shared decision making” per se (less than 1% of clinician utterances).

### Comparison with previous studies

While clinicians found coherence between their job and using the SDM conversation tool, they were less likely to agree that using the Anticoagulation Choice Decision Aid tool differed from their normal work. This is consistent with a metasynthesis of qualitative studies which found that for conversations about anticoagulation for atrial fibrillation, clinicians reported engaging in SDM routinely, even when patients were more likely to report a less participatory approach [14]. This was also supported by an evaluation of real-world distribution of decision aids; qualitative data suggested that clinicians did not have a shared understanding of the purpose of the decision aid [1].

Similar to the findings of the systematic review by Legaré et al., who documented that clinicians identify time constraints and lack of pertinence of the SDM intervention to patients and clinical situations as barriers to SDM implementation [5]. The design of the Anticoagulation Choice Decision Aid tool as an SDM conversation aid enables clinicians to tailor the tool to the patient’s situation [6], dealing with the latter barriers, and it may, in fact, efficiently guide a complex conversation and save time. Although SDM conversation tools demand dedicated and structured work that may change the way the clinician feels about time during the encounter, a recent systematic review of these tools demonstrated that, on average, these tools do not significantly prolong the clinical encounter [15].

Previous studies have also found burnout to be negatively associated with adaptive reserve [16], “practice features that enhance resilience, such as relationships” [17]. Burnout has also been associated with less empathic concern and decreased “effort to adopt the point of view of another person” [18]. While SDM has been proposed as an intervention to increase meaningful encounters and decrease burnout [19], our findings do not directly support an association between burnout and enthusiasm regarding SDM. However, given the highly positive response by clinicians to openness to collaboration with colleagues, their multiple inquiries regarding other clinicians using the Anticoagulation Choice Decision Aid tool, encouraging collaboration and innovation between team members, and involving local leadership may improve implementation of complex interventions such as SDM tools.

### Limitations

Process evaluations during trials may provide insights about the factors that promote or inhibit the implementation of complex interventions that are different from those gained from evaluations performed outside of research endeavors [2]. Research studies are temporary, require participant consent, and allow participant withdrawal without penalties (Fig. 1). Temporary add-on trial procedures, for instance, may overwhelm clinicians, who then opt out of the research, or, conversely, they may elicit weaker responses than permanent or mandatory practice changes. Similarly, clinicians choosing to participate in the trial may have been more enthusiastic about the intervention than those who declined. This study was conducted at the beginning of the trial, before participating clinicians had an opportunity to use the tool with patients. Therefore, we captured mostly clinicians’ expectations about using the SDM tool, informed by very brief training and demonstration when clinicians may have been more enthusiastic about the process. We did not assess “reflexive monitoring,” the fourth domain of NPT, which requires responding to questions after having regularly used the intervention, such as, “Given the changes observed as a result of the intervention, should we keep doing it?” For example, the organic monitoring, appraisal, and endorsement by clinical staff (represented by white arrows and white panel in Fig. 1) were not measured in our study. These limitations affect inferences about the normalization of SDM tools in practice to a greater extent than about the procedures for ongoing clinician recruitment into our trial, the purpose with which we designed this process evaluation. Finally, while participants were informed that the results would be anonymized, it is possible that clinicians may have felt uncomfortable reporting high levels of burnout for fear of stigma or professional repercussions.

### Implications for research and practice

It has been suggested that SDM may decrease clinician burnout by creating more meaningful patient–clinician encounters [19]. A clinician communication skills training intervention was associated with a significant and small decrease in burnout [20]. Yet, trials testing SDM tools rarely collect the clinician perspective, with no trials collecting measures of burnout or clinician well-being [15, 19, 21]. Our study explored the interplay between SDM implementation and contextual factors such as burnout and practice resources such as time in consultation. A key insight that participants offered and deserves further exploration is that the normalization of SDM tools in practice may benefit from supporting collective action. We found that people were enthusiastic about working with colleagues, a process hindered by their lack of awareness of who else was participating in it and who was driving the implementation of the tool in their clinics. Perhaps future research should ascertain not just clinician burnout but also isolation.

This process evaluation contributed to the rollout of the trial. Clinicians were invited to provide feedback and ask questions about trial procedures and the tool. When study staff (research assistants and study coordinators) were unable to answer questions in real time (e.g., about bleeding risk estimates), questions were referred to the principal investigators. The principal investigators, topic experts at their own sites, contacted participating clinicians to respond to these queries, communicating their buy-in in the use of the SDM tool. Internally, study staff maintained a live database of questions and topics that came up during clinician consent or NPT interviews. The study staff recorded and regularly updated crowdsourced answers (Fig. 1) to maintain coherence regarding the tool and trial within the study team and between study sites. In these interactions, we had to maintain a balance between facilitating the use of the SDM tool and maintaining a sense of uncertainty about its relative efficacy (rather than advocating for its value), which justified the clinical trial.

Clinicians made several requests to modify the content of the tool. For example, the tool was designed to support a conversation between clinicians and their patients; patient and clinician expertise and experience should enhance the tool and tailor its effect to each patient and situation, contributing to a pertinent and useful SDM conversation. In this way, we resisted adding more information to the tool, a common request, because this may have reduced clinician participation in the conversation and promoted instead an interaction between the patient and the SDM tool. Other changes could be accommodated. For example, we added the ability to toggle between 5-year and 1-year risk estimates.

Finally, an apparent disconnect emerged between the communication goals of the research and of clinicians, the former more focused on cocreation and the latter on conveying information. A previous study showed that even within clinic teams, there are dissimilar interpretations of SDM [22]. In our random sample of 30 recordings (112 clinicians), only 2 comments mentioned SDM, and these arose from making sense of using SDM tools rather than a philosophical discussion on the purpose of “shared decision making.” In this way, far from having arrived at a broad consensus of definition, purpose, and value of SDM, our study suggests that clinicians continue to negotiate the purpose and value of SDM in their practices. Future studies should continue to explore the clinician perspective, particularly when SDM implementation must take place within individual and collective workflows. Clinicians can both give meaning to SDM tools and preclude their presence in clinical encounters.

## Conclusions

We were able to use NPT to uncover potential barriers and facilitators to the use of an SDM conversation tool within a multicenter randomized trial evaluating its effectiveness. While most participants found SDM coherent with their clinical work and expressed high levels of buy-in, their engagement was dependent on the accuracy of its content and the feasibility of its use within busy clinical workflows. For some, using the SDM tool was perceived as a peripheral add-on, perhaps a desirable one, to their work rather than being integral to it. These views have implications for trial procedures and arduous implementation [23], were the trial to demonstrate that the SDM intervention contributes to care.

## Supplementary information

**Additional file 1.** Clinician baseline survey.

## Data Availability

The datasets used and/or analyzed during the current study are available from the corresponding author on reasonable request.
