# Supplementary material for: Normalization of a conversation tool to promote shared decision making about anticoagulation in patients with atrial fibrillation within a practical randomized trial of its effectiveness: a cross-sectional study
Source: Trials. 2020 May 12;21:395. doi: 10.1186/s13063-020-04305-2 (PMC7218532; doi:10.1186/s13063-020-04305-2)
Supplement: Supplementary file 1 — Additional file 1. Clinician baseline survey. [file 13063_2020_4305_MOESM1_ESM.doc]

**Appendix: Clinician Baseline Survey**

**Shared Decision Making for Stroke Prevention in Atrial Fibrillation**

**Clinician Study ID: __________________**

1. Today’s Date: __ __/__ __/__ __ __ __

Month Day Year

1. Are you: 1 ❑ Male 2 ❑ Female
2. In what year were you born? 1 9 ___ ___
3. What is your degree?

1 ❑ Nurse practitioner

2 ❑ Physician Assistant

3 ❑ Medical Doctor

4 ❑ Osteopathic Doctor

5 ❑ Pharmacist

6 ❑ Other, please specify: _______________________

1. Please indicate which of the following is your practice:

1 ❑ Family Medicine

2 ❑ Internal Medicine

3 ❑ Cardiology

4 ❑ Cardiac Electrophysiology

5 ❑ Pharmacy

6 ❑ Other, please specify: _________________________________________

1. Are you a resident/fellow? 1 ❑ Yes 2 ❑ No

If no, how many total years have you been in practice after completing all training (residency and fellowship)? ___ ___

1. How many years have you worked in this practice setting?

1 ❑ Less than one year

2 ❑ 1-4 years

3 ❑ 5-8 years

4 ❑ More than 8 years

1. In your estimation, how many patients do you evaluate per week who are considering or currently taking an anticoagulant? (Please provide a value rather than a range)

_________ Patients

**9**. I feel burned out from work.

|  |  |  |  |  |  |  |
| --- | --- | --- | --- | --- | --- | --- |
| Never | A few times a year or less | Once a month or less | A few times a month | Once a week | A few times a week | Every day |

**10.** I’ve become more callous toward people since I took this job.

|  |  |  |  |  |  |  |
| --- | --- | --- | --- | --- | --- | --- |
| Never | A few times a year or less | Once a month or less | A few times a month | Once a week | A few times a week | Every day |

**The following questions are designed to help get a better understanding of how to apply and integrate shared decision making interventions in health care. Please take the time to decide which answer best suits your experience for each statement and tick the appropriate response. This survey asks questions about anticoagulation choice decision aid.**

**11.** When you use the anticoagulation choice, how familiar does it feel? (Please mark one.)

0 1 2 3 4 5 6 7 8 9 10

Still feels very new Feels completely familiar

**12.** Do you feel the anticoagulation choice is currently a normal part of your work? (Please mark one)

0 1 2 3 4 5 6 7 8 9 10

No, not at all Yes, very much so

**13.** Do you feel the anticoagulation choice will become a normal part of your work? (Please mark one)

0 1 2 3 4 5 6 7 8 9 10

No, not at all Yes, very much so

**14.** For each statement below, please select an answer that best suits your experience using Option A. If the statement is not relevant to you, please select an answer from Option B.

|  | **Option A** | | | | |  | **Option B** | | |
| --- | --- | --- | --- | --- | --- | --- | --- | --- | --- |
|  | **Strongly agree** | **Agree** | **Neither agree or disagree** | **Disagree** | **Strongly disagree** |  | **Not relevant to my role** | **Not relevant at this stage** | **Not relevant to the intervention** |
| **a.** I can see how the anticoagulation choice differs from usual ways of working | 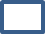 | 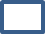 | 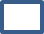 | 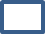 | 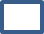 |  | 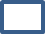 | 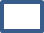 | 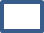 |
|  |  |  |  |  |  |  |  |  |  |
| **b.** Staff in this organization have a shared understanding of the purpose of the anticoagulation choice | 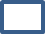 | 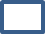 | 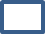 | 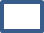 | 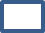 |  | 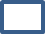 | 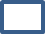 | 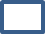 |
|  |  |  |  |  |  |  |  |  |  |
| **c.** I understand how the anticoagulation choice affects the nature of my own work | 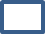 | 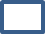 | 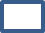 | 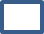 | 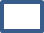 |  | 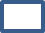 | 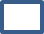 | 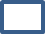 |
|  |  |  |  |  |  |  |  |  |  |
| **d.** I can see the potential value of the anticoagulation choice for my work | 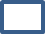 | 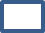 | 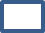 | 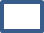 | 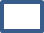 |  | 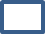 | 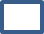 | 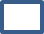 |
|  |  |  |  |  |  |  |  |  |  |
| **e.** There are key people who drive the anticoagulation choice forward and get others involved | 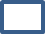 | 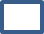 | 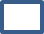 | 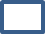 | 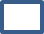 |  | 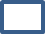 | 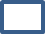 | 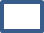 |
|  |  |  |  |  |  |  |  |  |  |
| **f.** I believe that participating in the anticoagulation choice is a legitimate part of my role | 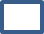 | 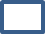 | 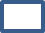 | 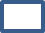 | 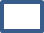 |  | 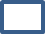 | 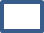 | 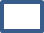 |
|  |  |  |  |  |  |  |  |  |  |
| **g.** I’m open to working with colleagues in new ways to use anticoagulation choice | 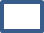 | 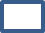 | 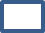 | 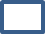 | 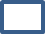 |  | 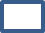 | 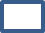 | 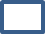 |
|  |  |  |  |  |  |  |  |  |  |
| **h.** I will continue to support anticoagulation choice | 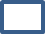 | 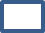 | 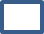 | 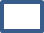 | 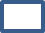 |  | 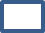 | 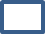 | 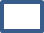 |
|  |  |  |  |  |  |  |  |  |  |
| **i.** I can easily integrate the anticoagulation choice into my existing work | 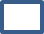 | 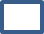 | 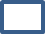 | 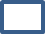 | 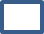 |  | 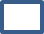 | 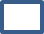 | 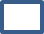 |
|  |  |  |  |  |  |  |  |  |  |
| **j.** Anticoagulation choice disrupts working relationships | 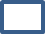 | 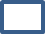 | 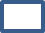 | 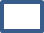 | 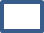 |  | 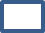 | 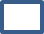 | 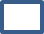 |
|  |  |  |  |  |  |  |  |  |  |
| **k.** I have confidence in other people’s ability to use anticoagulation choice | 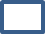 | 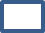 | 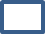 | 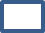 | 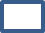 |  | 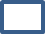 | 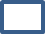 | 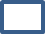 |
|  |  |  |  |  |  |  |  |  |  |
| **l.** Work is assigned to those with skills appropriate to anticoagulation choice | 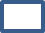 | 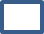 | 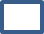 | 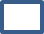 | 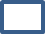 |  | 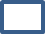 | 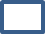 | 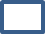 |
|  |  |  |  |  |  |  |  |  |  |
| **m.** Sufficient training is provided to enable staff to implement anticoagulation choice | 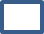 | 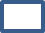 | 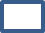 | 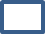 |  |  |  |  |  |
|  |  |  |  |  |  |  |  |  |  |
| **n.** Sufficient resources are available to support anticoagulation choice |  |  |  |  |  |  |  |  |  |
|  |  |  |  |  |  |  |  |  |  |
| **o.** Management adequately supports anticoagulation choice |  |  |  |  |  |  |  |  |  |
|  |  |  |  |  |  |  |  |  |  |
| **p.** I am aware of reports about the effects of anticoagulation choice |  |  |  |  |  |  |  |  |  |
|  |  |  |  |  |  |  |  |  |  |
| **q.** The staff agree that anticoagulation choice is worthwhile |  |  |  |  |  |  |  |  |  |
|  |  |  |  |  |  |  |  |  |  |
| **r.** I value the effects that anticoagulation choice has had on my work |  |  |  |  |  |  |  |  |  |
|  |  |  |  |  |  |  |  |  |  |
| **s.** Feedback about anticoagulation choice can be used to improve it in the future |  |  |  |  |  |  |  |  |  |
|  |  |  |  |  |  |  |  |  |  |
| **t.** I can modify how I work with anticoagulation choice |  |  |  |  |  |  |  |  |  |

**Thank you for your time. Please return the completed survey to the study coordinator.**
